# Supplementary material for: Dietary Environmental Footprints and Their Association with Socioeconomic Factors and Food Purchase Practices: BRAZUCA Natal Study
Source: Foods. 2022 Nov 28;11(23):3842. doi: 10.3390/foods11233842 (PMC9739984; doi:10.3390/foods11233842)
Supplement: Supplementary file 1 [file foods-11-03842-s001.zip › foods-1995732-supplementary.pdf]

Supplementary Material

Table of Contents

Table S1. *Per capita* consumption and EnF values estimated for each food group 2

Table S2. Adaptation of foods with no environmental footprint 3

Table S3. Statistical result of the correspondence analysis 5

References 5

Table S1. *Per capita* consumption and EnF values estimated for each food group

Food groups	Per capita (g/day)	CF (gCO_2eq/kg)	WF (L/kg)	EF (m^2/kg)
Milk	104.01	1,267.10	1,060.21	7.15
Yogurt	116.47	1,753.64	2,559.34	24.74
Cheese	29.89	8,734.05	6,491.87	33.73
Bread	51.12	833.67	1,683.72	6.02
Cookies	33.84	1,558.91	1,899.90	11.02
Cake	88.25	2,286.91	2,140.38	24.34
Breakfast cereal, oatmeal, granola	9.88	1,364.02	2,560.53	7.10
Coffee	134.71 mL (6.74 g – 5% of the liquid)	372.57	12,150.61	51.58
Tea	160.73 mL (2.01 g – 1.25% of the liquid)	101.86	1,738.95	11.04
Potato, cassava, polenta	65.58	477.92	909.86	4.60
Farofa*	14.40	610.63	1,692.33	8.86
Couscous and tapioca	51.87	716.08	1,488.47	9.31
Beans	70.47	353.62	1,421.16	3.88
Rice	59.71	1,062.38	648.03	5.29
Eggs	45.40	3,760.88	3,420.73	23.52
Jerked or sun-dried beef	29.89	49,769.92	46,567.06	35.26
Beef	79.24	42,353.44	28,424.13	176.64
Sausage	26.50	25,805.68	22,556.94	75.42
Pork	37.11	7,840.74	8,226.98	57.06
Chicken	63.60	5,224.04	6,643.92	39.00
Fish	98.98	5,462.13	1,438.83	311.04
Seafood	59.69	56,165.44	61.33	565.15
Cold cuts	27.34	8,924.22	10,987.98	41.71
Noodles, lasagna, gnocchi	64.23	1,614.25	1,333.25	8.19
Fried savory pastries	40.45	8,447.73	1,730.20	10.50
Baked savory pastries	204.99	8,558.92	1,672.70	10.37
Pizza	20.99	4,400.00	1,270.21	9.44
Greeneries	7.48	367.60	646.50	2.09
Tomato	20.62	881.71	420.15	3.53
Legumes	6.52	342.77	2,269.67	1.78
Fruits	106.78	954.20	1,658.58	6.10
Snacks	47.60	3,900.00	1,270.21	9.44
Peanuts, nuts, walnuts	14.80	2,501.21	18,914.81	8.88
Sweetener	0.25	11,564.11	19,092.31	17.51
Sugar	12.37	495.70	706.06	3.78
Industrialized juice	230.06	477.70	1,518.85	57.50
Natural juice	241.46	243.41	300.17	7.03
Soda	248.38	744.48	450.00	2.00
Chocolate or bonbons	11.91	2,433.00	23,958.45	43.75
Confectionary sweets	50.32	2,194.77	1,993.39	12.23
Ice cream and milkshake	112.88	2,350.00	6,114.91	33.50

* A side dish prepared with cassava or corn flour, usually roasted, seasoned, and sautéed with vegetable, lard, or butter added with eggs, bacon, jerked beef, olives, or other ingredients

Table S2. Adaptation of foods with no environmental footprint

Food with no footprint (24HR) [1]	Adaptation/Equivalent [2]
Petit Suisse cheese<br>Plum yogurt	Yogurt of any flavor
Cheddar cheese	Unspecified cheese
Parmesan cheese	Ground cheese
Garlic bread<br>Italian bread<br>Pita bread	Bread roll
Industrialized milk bread<br>Traditional sliced bread sandwich with salted butter	Industrialized loaf bread of any brand
Hot dog bun	Hamburger bun
Brown loaf bread	Brown bread
Bread roll sandwich	Bread roll
Brown toast<br>Traditional toast	Toast of any bread
Rice cracker	Unspecified cracker
Cookie, sweet, shortbread<br>Cookie, sweet, shortbread, chocolate<br>Cookie, sweet, shortbread, coconut<br>Cookie, sweet, banana/oat/honey<br>Cookie, sweet, ladyfinger<br>Cookie, sweet, chocolate, w/ 5 grains<br>Cookie, sweet, cornstarch<br>Cookie, sweet, Marie<br>Cookie, sweet, simple (average of different types)	Sweet cookie
Cookie, savory (average of different brands and types)<br>Cracker, savory, water<br>Cracker, savory, cream cracker<br>Cracker, savory, cream cracker, whole-grain	Savory cookie
Cake, vanilla, strawberry-flavored filling<br>Cake, industrialized(average of different flavors)<br>Cake, wheat, simple, homemade	Wheat cake
Cake, corn meal, homemade	Corn cake
Chia seed	Flax seed
Percolated coffee<br>Instant coffee<br>Decaffeinated coffee	Coffee
Sautéed cassava	Braised cassava
Hump steak<br>Outside flat<br>Topside	Beef
Main cuts (different preparations)<br>Secondary cuts (different preparations)<br>Varied barbecue skewer	Beef
Braised knuckle	Braised beef
Whole free-range chicken	Chicken
Braised chicken heart	Grilled chicken heart
Chicken leg	Chicken cuts

Chicken thigh	
Prepared chicken meat	Unspecified chicken part
Whole-grain noodles	Noodles
Shortcut pastry<br>Oven pastry	Savory pie
Puff pastry	Croissant
Parsley and chives<br>Parsley	Coriander
Basil	Mint
Raw vegetable salad<br>Salad, white cabbage, with vinaigrette sauce	Salad or cooked greenery, except fruit salad
Blueberry	Blackberry
Date	Plum
Beverage, powdered juice, prepared, w/ water<br>Juice, concentrate, prepared, cashew, w/ sugar<br>Juice, concentrate, prepared, cashew, no sugar<br>Juice, concentrate, prepared, grape, w/ sugar<br>Grape, juice, concentrate, bottled	Beverage
Tamarind, watermelon, hog plum	Juice
Lime juice	Orange juice
Chocolate cream<br>Bittersweet chocolate<br>Milk chocolate	Chocolate
Soda, w/ sweetener (average of different flavors)<br>Soda, regular, w/ sugar (average of different samples)	Unspecified soda

Table S3. Statistical result of the correspondence analysis

**Independence test between rows and columns**			
	CF	WF	EF
Chi-squared (observed value)	85.877	80.233	44.147
Chi-squared (critical value)	69.832	69.832	69.832
DF	52	52	52
p-value	0.002	0.007	0.772
alpha	0.05	0.05	0.05

Test interpretation:

H0: The table rows and columns are independent.

Ha: There is a dependence between the table rows and columns.

Since the calculated p-value is lower than the significance level alpha=0.05, the H0 null hypothesis must be rejected in favor of the alternative hypothesis Ha.

The risk of rejecting the H0 null hypothesis when it is true is below 0.01%.

References

1. Universidade de São Paulo (USP).; Food Research Center (FoRC). Tabela Brasileira de Composição de Alimentos (TBCA). 2020.
2. Garzillo, J.M.F.; Machado, P.P.; Louzada, M.L. da C.; Levy, R.B.; Monteiro, C.A. *Pegadas Dos Alimentos e Das Preparações Culinárias Consumidos No Brasil*; Universidade de São Paulo. Faculdade de Saúde Pública, 2019; ISBN 9788588848368.
